# Gap junctions and hemichannels composed of connexins: potential therapeutic targets for neurodegenerative diseases

**DOI:** 10.3389/fncel.2014.00189

**Published:** 2014-09-02

**Authors:** Hideyuki Takeuchi, Akio Suzumura

**Affiliations:** Department of Neuroimmunology, Research Institute of Environmental Medicine, Nagoya UniversityNagoya, Japan

**Keywords:** glutamate, microglia, neuroinflammation, neurodegeneration, gap junction, hemichannel, connexin

## Abstract

Microglia are macrophage-like resident immune cells that contribute to the maintenance of homeostasis in the central nervous system (CNS). Abnormal activation of microglia can cause damage in the CNS, and accumulation of activated microglia is a characteristic pathological observation in neurologic conditions such as trauma, stroke, inflammation, epilepsy, and neurodegenerative diseases. Activated microglia secrete high levels of glutamate, which damages CNS cells and has been implicated as a major cause of neurodegeneration in these conditions. Glutamate-receptor blockers and microglia inhibitors (e.g., minocycline) have been examined as therapeutic candidates for several neurodegenerative diseases; however, these compounds exerted little therapeutic benefit because they either perturbed physiological glutamate signals or suppressed the actions of protective microglia. The ideal therapeutic approach would hamper the deleterious roles of activated microglia without diminishing their protective effects. We recently found that abnormally activated microglia secrete glutamate via gap-junction hemichannels on the cell surface. Moreover, administration of gap-junction inhibitors significantly suppressed excessive microglial glutamate release and improved disease symptoms in animal models of neurologic conditions such as stroke, multiple sclerosis, amyotrophic lateral sclerosis, and Alzheimer's disease. Recent evidence also suggests that neuronal and glial communication via gap junctions amplifies neuroinflammation and neurodegeneration. Elucidation of the precise pathologic roles of gap junctions and hemichannels may lead to a novel therapeutic strategies that can slow and halt the progression of neurodegenerative diseases.

## Introduction

Microglia are macrophage-like immune cells that reside in the central nervous system (CNS), where they play multiple roles: presenting antigen to initiate immunological reactions, directing attack against non-self antigens, debris clearance, support of neuronal circuit development (Kreutzberg, 1996; Kempermann and Neumann, 2003; Block et al., 2007; Takeuchi, 2010; Boche et al., 2013), and so on. Microglia contribute to maintenance of CNS homeostasis, but abnormal activation of these cells often causes damage to surrounding cells and tissues. Microgliosis, the accumulation of activated microglia, is a characteristic pathological feature in many neurologic conditions such as trauma, stroke, inflammation, epilepsy, and neurodegenerative diseases (Cagnin et al., 2001; Eikelenboom et al., 2002; McGeer and McGeer, 2002; Nelson et al., 2002; Orr et al., 2002; Bruijn et al., 2004; Pavese et al., 2006). Activated microglia release massive amounts of glutamate, at much higher levels than astrocytes and neurons (mM vs. μM), and destroy neural cells; these processes have been implicated as a major cause of neuronal damage in neurologic diseases (Piani et al., 1992; Barger and Basile, 2001; Schwartz et al., 2003; Ye et al., 2003; Kipnis et al., 2004; Takeuchi et al., 2005, 2008b; Herman and Jahr, 2007; Liang et al., 2008; Yawata et al., 2008). Therefore, blockade of glutamate signaling and inhibition of microglial activation have been explored as therapeutic candidates for several neurodegenerative diseases. However, glutamate receptor blockers also perturb physiological glutamate signals and cause severe adverse side effects (Parsons et al., 2007). Tetracycline and two of its derivatives (doxycycline and minocycline) have been used as inhibitors of microglial activation, but these compounds exerted little therapeutic benefit, because activated microglia also exert neuroprotective effects such as production of neurotrophic factors and sequestration of neurotoxic substances (Zietlow et al., 1999; Kempermann and Neumann, 2003; Kipnis et al., 2004; Koenigsknecht and Landreth, 2004; Schwab and Schluesener, 2004). Thus, the optimal therapeutic strategy would inhibit the deleterious effects of activated microglia without diminishing their protective roles (Takeuchi, 2010). We recently found that neurotoxic activated microglia secrete glutamate through gap-junction hemichannels. Recent evidence also suggests that neuronal and glial communication by gap junctions amplifies neuroinflammation and neurodegeneration. Therefore, elucidation of the pathologic roles of gap junctions and hemichannels may provide us with new therapeutic strategies against many neurologic diseases.

## Microglia as the “enemy within”

Microglia, which originate from bone marrow–derived myeloid cells, account for approximately 10% of cells in the human CNS (Del Rio-Hortega, 1932). Microglia are predominantly observed in gray matter, especially in the olfactory bulb, hippocampus, basal ganglia, and substantia nigra (Lawson et al., 1990). Under healthy physiological conditions, microglia persist in a quiescent state with ramified morphology (resting microglia) and survey the environment of the CNS (Davalos et al., 2005; Nimmerjahn et al., 2005). Under pathological conditions, microglia dramatically change their morphology and adopt an amoeboid appearance in the activated state. Activated microglia express surface molecules such as Fc receptor, CD11b, CD11c, CD14, major histocompatibility complex (MHC) molecules, Toll-like receptors (TLRs), scavenger receptors, and cytokine/chemokine receptors, and they can act as both antigen-presenting cells and immunological effector cells (Suzumura et al., 1987; Rock et al., 2004). In addition to innate immunity, activated microglia also play other beneficial roles, such as neuroprotection mediated by release of neurotrophic factors (Zietlow et al., 1999; Bessis et al., 2007; Liang et al., 2010), maintenance of CNS homeostasis by clearance of cellular debris and toxic substances (Upender and Naegele, 1999; Marin-Teva et al., 2004; Iribarren et al., 2005; Simard et al., 2006; Richard et al., 2008), and guidance of stem-cell migration in neuronal repair and neurogenesis (Aarum et al., 2003; Ziv et al., 2006a,b).

TLRs and scavenger receptors may contribute to diminishing neurotoxicity by sequestering neurotoxic substances such as amyloid β (El Khoury et al., 1996; Coraci et al., 2002; Bamberger et al., 2003; Liu et al., 2005); however, signals downstream of these receptors also enhance microglial neurotoxic effects by producing neurotoxic factors such as cytokines/chemokines, nucleic acids, excitatory amino acids, reactive oxygen species (ROS), and proteases (Kempermann and Neumann, 2003; Takeuchi et al., 2005, 2006; Kawanokuchi et al., 2006; Block et al., 2007). In fact, expression levels of TLRs and scavenger receptors are upregulated in a variety of neurologic diseases (Akiyama and McGeer, 1990; Grewal et al., 1997; El Khoury et al., 1998; Bsibsi et al., 2002; Cho et al., 2005; Carpentier et al., 2008). Therefore, whether activated microglia exert a neurotoxic or a neuroprotective effect may depend on their environment, the spatiotemporal distribution of the microglia themselves, and the type and magnitude of stimuli (Jimenez et al., 2008; Wu et al., 2008; Nakanishi and Wu, 2009; Sawada, 2009). A recently proposed hypothesis suggests, by analogy to macrophage activation, that activated microglia comprise two subpopulations, the neurotoxic (M1) and neuroprotective (M2) species (Mantovani et al., 2002; Henkel et al., 2009; Boche et al., 2013); however, this hypothesis is still open to debate. At least in pathological conditions, deleterious microglial activation is probably involved in the progression of various neurological disorders (Yrjanheikki et al., 1998; Wu et al., 2002; Zhu et al., 2002; Stirling et al., 2004; Boillee et al., 2006; Seabrook et al., 2006). Thus, elucidating the precise mechanism of microglial neurotoxicity is a necessary step toward development of effective therapeutic strategies against neurologic diseases.

## Glutamate as a major neurotoxic factor from microglia

Glutamate is the most potently neurotoxic factor released from activated microglia. Excessive glutamate induces severe neuronal damage via excitotoxicity (Piani et al., 1992; Barger and Basile, 2001; Takeuchi et al., 2005, 2006). A common misconception is that inflammatory cytokines produced by activated microglia directly induce neuronal damage. In fact, these cytokines have little direct neurotoxic effect (Takeuchi et al., 2006; Takeuchi, 2010). Although tumor necrosis factor-α (TNF-α) and interferon-γ (IFN-γ) are considered to be the most deleterious inflammatory cytokines produced by activated microglia, these cytokines have only weak direct neurotoxic effects because they also enhance neuroprotective cascades involving mitogen-activated protein kinase (MAPK) and expression of nuclear factor κB (NF-κB) (Ghezzi and Mennini, 2001; Kamata et al., 2005). In general, inflammatory cytokines induce neurotoxicity indirectly by stimulating microglia in an autocrine/paracrine manner. These stimuli induce microglia to release high levels of glutamate, resulting in neuronal damage via excitotoxicity. Moreover, a recent paper showed that activated microglial glutamate suppresses astrocytic glutamate transporters, which play a pivotal role in maintenance of the physiological extracellular glutamate level (Takaki et al., 2012); this suppression probably worsens excitotoxic neuronal damage. Although microglia also express glutamate transporters, they seem much less effective at maintaining extracellular glutamate homeostasis than astrocytic glutamate transporters (Liang et al., 2008).

One of the earliest pathologic features of excitotoxicity is formation of neuritic beading, i.e., focal bead-like swelling in dendrites and axons (Takeuchi et al., 2005; Mizuno et al., 2008). Neuritic beading is a common neuropathological hallmark of many neurologic conditions such as ischemia, epilepsy, mechanical pressure, brain tumor, aging, neuroinflammatory diseases, and neurodegenerative diseases such as multiple sclerosis (MS), Alzheimer's disease (AD), Parkinson's disease (PD), and amyotrophic lateral sclerosis (ALS) (Delisle and Carpenter, 1984; Hori and Carpenter, 1994; Takahashi et al., 1997; Trapp et al., 1998; Dickson et al., 1999; Mattila et al., 1999; Swann et al., 2000; Goel et al., 2003; Pavlidis et al., 2003; Saito et al., 2003; Dutta and Trapp, 2007). Recent studies elucidated the detailed role of microglial glutamate in formation of neuritic beading and subsequent neuronal death. Glutamate produced by activated microglia activates neuronal N-methyl-D-aspartate (NMDA) receptor signaling, which promotes Ca^2+^ influx and activates Ca^2+^/calmodulin-dependent protein kinase (CaMK). CaMK activates neuronal nitric oxide synthase (nNOS) and increases the intracellular concentration of nitric oxide (NO). NO in turn inhibits mitochondrial respiratory chain complex IV, resulting in a rapid reduction in intracellular ATP levels. Ultimately, the loss of intracellular energy pools suppresses dendritic and axonal transport, leading to bead-like accumulation of cytoskeletal and motor proteins along neurites and the formation of neuritic beading. Thus, a low-energy state results in neuronal dysfunction. Persistence of this neuronal dysfunction eventually causes neuronal death [i.e., excitotoxic neuronal death or non-cell-autonomous neuronal death (Lobsiger and Cleveland, 2007)].

**Figure 1:**
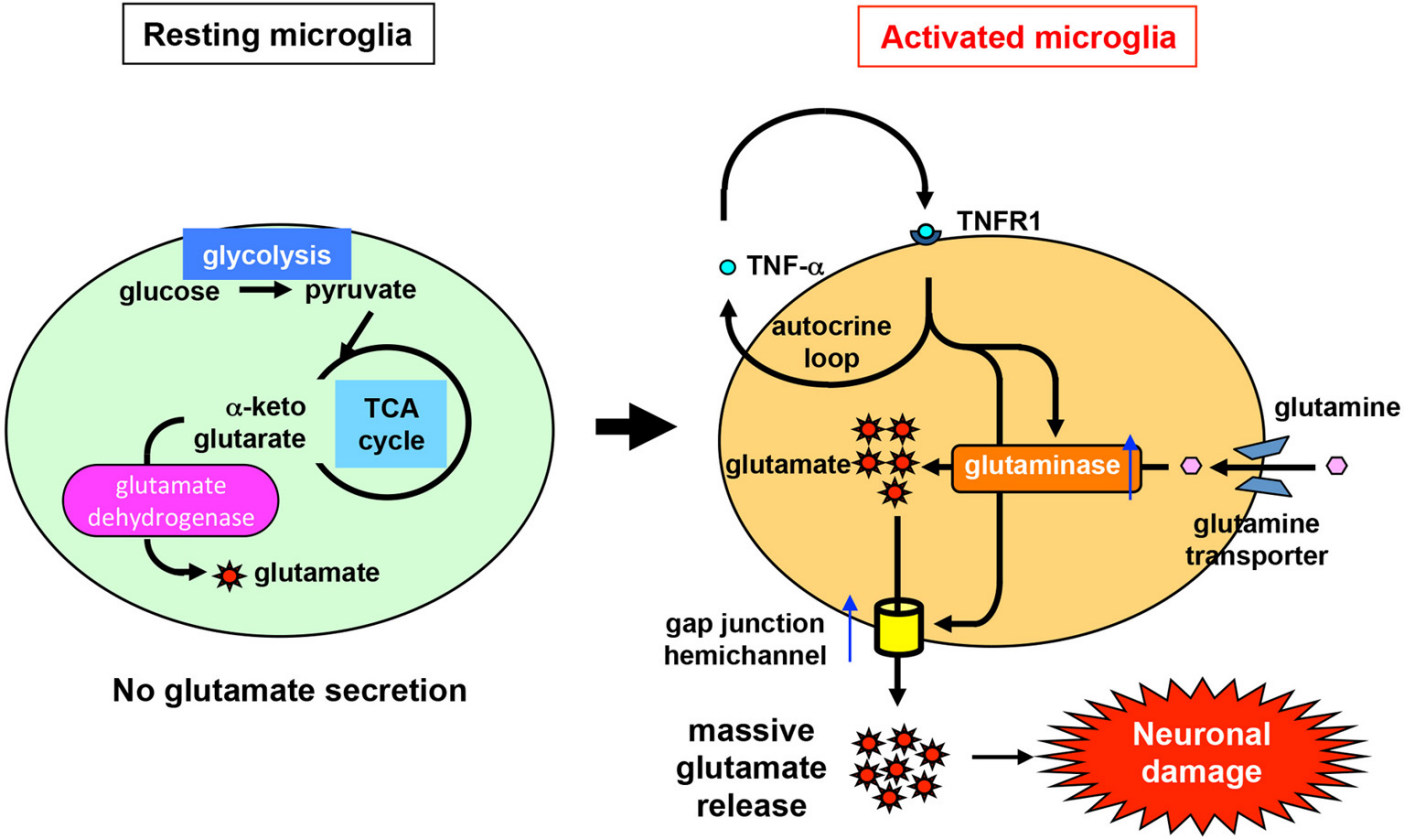
**Mechanism of glutamate production and release by activated microglia**. Like other types of cells, resting microglia use glutamate dehydrogenase to synthesize glutamate from intracellular α-ketoglutarate in order to maintain a physiologically normal level of glutamate. Under resting conditions, microglia release very little glutamate into the extracellular space. By contrast, under pathological conditions, glutaminase and gap-junction hemichannels are upregulated in activated microglia (e.g., in response to stimulation by TNF-α). Glutaminase synthesizes excess glutamate from extracellular glutamine, which is brought into the cell via glutamine transporters. Subsequently, high levels of glutamate are secreted through gap-junction hemichannels, resulting in eventual neuronal damage.

Recent studies have revealed the precise mechanism of glutamate production by activated microglia (Takeuchi et al., 2005, 2006) (Figure 1). Two pathways are involved in cellular glutamate synthesis (Newsholme and Newsholme, 1989; Newsholme and Calder, 1997; Yudkoff, 1997; Nissim, 1999). One of these pathways is mediated by glutamate dehydrogenase, which converts α-ketoglutarate to glutamate. Most cells use this pathway to maintain cellular homeostasis of glutamate levels. The other pathway is mediated by glutaminase, which produces glutamate from extracellular glutamine brought into the cell via glutamine transporters. Resting microglia maintain their physiological glutamate level via the glutamate dehydrogenase pathway, as in other cell types, and secrete very little glutamate into the extracellular space (Figure 1). By contrast, activated microglia produce excessive amounts of glutamate as a result of upregulation of glutaminase, but not glutamate dehydrogenase. Subsequently, activated microglia release massive amounts of glutamate via gap-junction hemichannels. Inflammatory cytokines such as TNF-α and IFN-γ enhance not only glutaminase expression level but also cell-surface localization of hemichannels in microglia (Eugenin et al., 2001; Takeuchi et al., 2006). These two phenomena may act synergistically to release excess glutamate, leading to excitotoxic neuronal damage (Figure 1). Moreover, the extracellular glutamine level is critical for microglial glutamate production (Takeuchi et al., 2006). In the CNS, glutamine from astrocytes is essential for glutamate production in neurons (Tsacopoulos and Magistretti, 1996), suggesting that it also plays an important role in microglial glutamate production.

## Gap junctions in CNS cells

**Figure 2:**
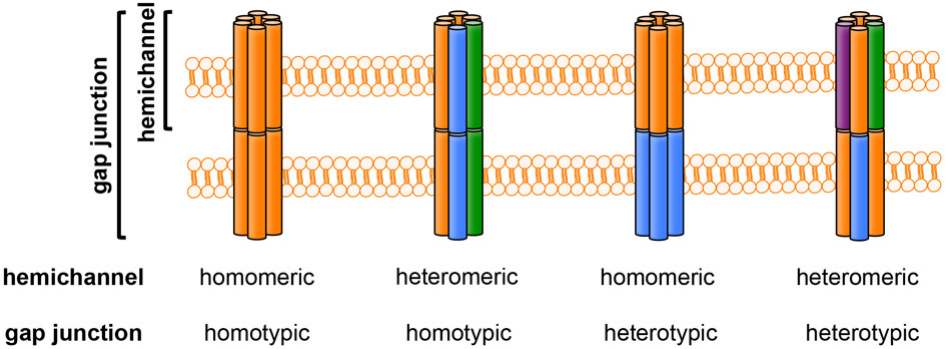
**The composition of gap junctions and hemichannels**. Each colored column (orange, blue, green, or purple) represents a different connexin isoform. Hemichannels may be homomeric (composed of one connexin isoform) or heteromeric (composed of more than one connexin isoform). Gap-junction channels may be homotypic (formed by identical hemichannels) or heterotypic (formed by different hemichannels).

Gap junctions contribute to formation of intercellular channels that directly connect the cytoplasmic compartments of neighboring cells (Yeager and Harris, 2007). These channels pass various small molecules (~1000 Da) and ions, although the charges and shapes of these molecules may affect the rate of transfer through gap junctions (Goldberg et al., 2004). Each gap junction is composed of a pair of hemichannels docked in a head-to-head configuration. Hemichannels are organized as hexagonal cylinders with central pores, and each hemichannel consists of a hexameric cluster of protein subunits called connexins (in vertebrates) or innexins (in invertebrates). Connexins are encoded by a conserved family of genes with at least 21 members in mammals. There are 21 connexin genes in the human genome and 20 connexin genes in the mouse genome; 19 of these proteins have orthologs in both humans and mice (Willecke et al., 2002; Laird, 2006). The connexin isoforms structurally interact in multiple ways. Homomeric hemichannels consist of a single connexin isoform, whereas heteromeric hemichannels contain two or more different connexin isoforms. Likewise, a homotypic gap junction channel is composed of two identical hemichannels, whereas a heterotypic gap junction channel contains two different hemichannels. Thus, the compositions of gap junctions can be classified into four types: homomeric and homotypic; heteromeric and homotypic; homomeric and heterotypic; and heteromeric and heterotypic (Figure 2). This heterogeneity of connexin configurations confers complexity to the gap junction/hemichannel system.

Gap junctions allow direct intracellular propagation of second messengers (e.g., Ca^2+^, IP_3_, cAMP, and cGMP), metabolites (e.g., glutamate, glucose, and glutathione), and nucleotides (e.g., ATP, ADP, and RNA) between adjacent cells (Goldberg et al., 1999, 2002; Harris, 2001, 2007; Saez et al., 2003; Valiunas et al., 2005; Laird, 2006). Moreover, recent studies revealed that uncoupled “free” hemichannels facilitate two-way transfer of molecules between the cytosol and extracellular milieu (De Vuyst et al., 2007; Retamal et al., 2007; Laird, 2010). Intracellular communication via gap junctions and hemichannels is regulated by such mechanisms as channel gating via chemicals, pH, and voltage, as well as by changes in connexin transcription, translation, post-translational phosphorylation and ubiquitination, membrane insertion, and hemichannel internalization and degradation (Laird, 2006; Leithe and Rivedal, 2007; Solan and Lampe, 2009). The time courses of these changes range from milliseconds to hours and are influenced by the environmental conditions in cells and tissues.

Whereas vertebrate cells use connexins to form gap junctions and hemichannels, invertebrate cells use innexins, which lack sequence homology to connexins. A search of the human genome identified three innexin-related genes (Barbe et al., 2006). Because of the occurrence of homologous genes in both vertebrates and invertebrates, the corresponding proteins were termed pannexins: pannexin1 (Panx1), pannexin2 (Panx2), and pannexin3 (Panx3). Pannexins have the same transmembrane topology as connexins, with four transmembrane domains and cytoplasmic amino-terminal and carboxyl-terminal domains. Recent evidence indicates that pannexins also form uncoupled hemichannels in mammalian cells; however, it is not clear whether they can form functional gap junctions (Dahl and Locovei, 2006).

Tissues have characteristic connexin expression profiles, and neural cells in the CNS express multiple connexins (Dermietzel et al., 1989, 2000; Bittman and Loturco, 1999; Chang et al., 1999; Nagy and Rash, 2000; Eugenin et al., 2001; Rash et al., 2001; Teubner et al., 2001; Altevogt et al., 2002; Parenti et al., 2002; Rouach et al., 2002; Odermatt et al., 2003; Takeuchi et al., 2006). All neurons express Cx36 and Cx45, whereas other neural connexins are expressed with more specific spatiotemporal profiles (Sohl et al., 2005). Electrical coupling between neurons has been implicated in neuronal synchronization in the CNS (Christie et al., 1989; Bouskila and Dudek, 1993; Wong et al., 1995). Neuronal gap junctions composed of Cx36 and Cx45 are thought to be homomeric and homotypic (Al-Ubaidi et al., 2000; Teubner et al., 2001), and these junctions are important for formation of electrical synapses (Deans et al., 2001; Hormuzdi et al., 2001). Rodent knockout models have shown that other connexins can compensate for the functions of Cx36 and Cx45, despite differences in conformation or permeability (Frank et al., 2010; Zlomuzica et al., 2010). Furthermore, there is accumulating evidence that gap-junction coupling plays a pivotal role in neuronal differentiation. Mice lacking Cx43 exhibit neonatal death and abnormal migration in the neural crest and neocortex (Lo et al., 1999; Xu et al., 2001; Fushiki et al., 2003). Blockade of gap junctions also hampers retinoic acid–induced neuronal differentiation of NT2 and P19 cells (Bani-Yaghoub et al., 1999a,b). Moreover, Cx36-containing gap junctions are important in neuronal remodeling and short-term spatial memory in some mature organisms (Allen et al., 2011; Hartfield et al., 2011). In contrast to neuron–neuron coupling, for which the evidence is convincing, the existence of functional neuron–glia coupling in the CNS is still a matter of debate (Nadarajah et al., 1996; Alvarez-Maubecin et al., 2000; Rash et al., 2001, 2007).

Astrocytes, the main CNS cells coupled via gap junctions, primarily express Cx43 and Cx30 (Dermietzel et al., 1991; Nagy and Rash, 2000). Consistent with this, Cx43/Cx30 double-knockout mice exhibit minimal gap-junction communication between astrocytes (Wallraff et al., 2006; Rouach et al., 2008), suggesting that functional astrocytic gap junctions are composed predominantly of these two connexins. Cx43-deficient astrocytes exhibit reduced gap-junction coupling, although they express other connexin subtypes including Cx30, Cx26, Cx40, Cx45, and Cx46 (Naus et al., 1997; Scemes et al., 1998; Dermietzel et al., 2000). Although Cx30 has been detected exclusively in astrocytes, Cx30 knockout mice develop only mild abnormalities, including hearing loss due to cochlear degeneration (Teubner et al., 2003). Thus, other astrocytic connexin subtypes do not seem to compensate for a lack of Cx43. Astrocytic gap junctions facilitate the formation of functional syncytium that buffers extracellular glutamate elevation, pH, and K^+^ concentrations associated with firing neurons, and also propagates intracellular Ca^2+^ waves that modulate neuronal activities (Walz and Hertz, 1983; Jefferys, 1995; Charles, 1998; Anderson and Swanson, 2000; Ransom et al., 2003). Moreover, astrocytic gap-junction communication facilitates trafficking of glucose and its metabolites, thereby mediating interactions between cerebral vascular endothelium and neurons (Giaume et al., 1997; Goldberg et al., 1999; Tabernero et al., 2006). Thus, astrocytic gap junctions play pivotal roles in modulating neuronal activities and maintaining CNS homeostasis. Astrocyte–astrocyte coupling can be achieved by any of the allowed combinations of homomeric or heteromeric hemichannels in homotypic or heterotypic configurations. Cx30 and Cx26 form both heteromeric and heterotypic channels (Nagy et al., 2003; Altevogt and Paul, 2004), whereas Cx43 forms homomeric and homotypic channels (Orthmann-Murphy et al., 2007). A previous report demonstrated that gap-junction coupling in astrocytes results in two distinct subpopulations of cells. Astrocytes expressing glutamate transporters are extensively coupled to each other, whereas strocytes expressing glutamate receptors are not coupled to other astrocytes (Wallraff et al., 2004), suggesting that these cells play a role in buffering extracellular glutamate (Anderson and Swanson, 2000).

Oligodendrocytes primarily express Cx29, Cx32, and Cx47 (Dermietzel et al., 1989; Altevogt et al., 2002; Odermatt et al., 2003). Oligodendrocytic gap junctions facilitate the trafficking of ions and nutrients from somas to myelin layers (Paul, 1995). Mice lacking Cx32 exhibit reduced myelin volume, enhanced excitability in the CNS, and progressive peripheral neuropathies (Anzini et al., 1997; Sutor et al., 2000). Cx32/Cx47 double-knockout mice develop abnormal movements and seizures associated with vacuolated myelin and axonal degeneration in the CNS, whereas Cx47-deficient mice exhibit only minimal CNS abnormalities (Menichella et al., 2003). Cx32 and Cx47 in oligodendrocytes are essential for spatial buffering of K^+^ in response to neuronal activity; failure of this function leads to myelin swelling and subsequent axonal degeneration (Menichella et al., 2006). Oligodendrocyte–oligodendrocyte coupling is mediated by gap junctions in homotypic configurations with homomeric or heteromeric hemichannels containing Cx32 or Cx47 (Orthmann-Murphy et al., 2007). Furthermore, oligodendrocytes also couple with astrocytes. Astrocyte–oligodendrocyte coupling may include heterotypic configurations of Cx43–Cx47, Cx30–Cx32, or Cx26–Cx32 (Nagy et al., 2003; Altevogt and Paul, 2004; Orthmann-Murphy et al., 2007). In addition to astrocyte–astrocyte coupling, astrocyte–oligodendrocyte coupling is important in the glial syncytium to facilitate the propagation of Ca^2+^ waves and the buffering of extracellular K^+^ and neurotransmitters such as glutamate (Walz and Hertz, 1983; Jefferys, 1995; Charles, 1998; Anderson and Swanson, 2000; Ransom et al., 2003).

Microglia express Cx32, Cx36, and Cx43 (Eugenin et al., 2001; Parenti et al., 2002; Garg et al., 2005; Takeuchi et al., 2006; Kielian, 2008; Talaveron et al., 2014), but form few functional gap junctions under resting conditions. The expression of connexins rises in activated microglia, although it remains unclear whether upregulated expression of connexins leads to enhanced formation of functional gap junctions with microglia and other CNS cells (Eugenin et al., 2001; Garg et al., 2005; Kielian, 2008; Takeuchi, 2010; Wasseff and Scherer, 2014). Recent evidence demonstrates that uncoupled microglial hemichannels play important roles in bidirectional trafficking of small molecules between the cytoplasm and extracellular space (Takeuchi et al., 2011; Eugenin et al., 2012).

The evidence described above might give the false impression that CNS cells express only a narrow range of combinations of homomeric hemichannels and gap junctions. However, the precise configuration of these hemichannels [i.e., homomeric and homotypic; heteromeric and homotypic; homomeric and heterotypic; and heteromeric and heterotypic (Figure 2)] has yet to be elucidated. In addition, our recent reverse transcription–PCR analysis using mouse primary cultures indicated that gap junctions/hemichannels in neurons and glial cells may consist of a wider range of combinations of connexins than expected (Table 1) (Takeuchi et al., 2014): neurons predominantly express Cx43, Cx50, Cx31, Cx30.3, Cx29, and Cx36; microglia predominantly express Cx46, Cx37, Cx40, Cx33, Cx57, Cx32, Cx31, Cx30.3, Cx47, Cx36, Cx30.2, Cx39, and Cx23; astrocytes predominantly express Cx43, Cx37, Cx57, Cx26, Cx31, Cx30.3, Cx45, Cx30.2, Cx39, and Cx23; and oligodendrocytes predominantly express Cx46, Cx37, Cx40, Cx33, Cx57, Cx32, Cx26, Cx31, Cx30.3, Cx31.1, Cx30, Cx29, Cx36, Cx30.2, Cx39, and Cx23. These findings imply that connexin expression profiles in the CNS are dynamic, both in healthy and pathological states.

**Table 1:** **mRNA expression levels of mouse connexin in CNS cells**.

**Mouse**		**mRNA expression level**			
**Protein**	**Gene**	**Neuron**	**Microglia**	**Astrocyte**	**Oligodendrocyte**
Cx43	*Gja1*	++	±	+++	+
Cx46	*Gja3*	+	+++	+	++
Cx37	*Gja4*	+	+++	++	+++
Cx40	*Gja5*	+	+++	+	+++
Cx33	*Gja6*	+	++	+	++
Cx50	*Gja8*	+++	+	±	+
Cx57	*Gja10*	±	+++	+++	+++
Cx32	*Gjb1*	+	+++	+	+++
Cx26	*Gjb2*	+	+	+++	+++
Cx31	*Gjb3*	++	++	++	++
Cx30.3	*Gjb4*	++	++	++	++
Cx31.1	*Gjb5*	+	+	+	+++
Cx30	*Gjb6*	±	+	+	+++
Cx45	*Gjc1*	−	+	+++	±
Cx47	*Gjc2*	±	+++	+	+
Cx29	*Gjc3*	++	+	±	++
Cx36	*Gjd2*	+++	++	−	++
Cx30.2	*Gjd3*	+	++	++	+++
Cx39	*Gjd4*	±	++	++	+++
Cx23	*Gje1*	±	++	++	+++

*−, none; ±, slight; +, low; ++, moderate; +++, high*.

## Gap junctions composed of connexins as a novel therapeutic target for neurologic diseases

**Figure 3:**
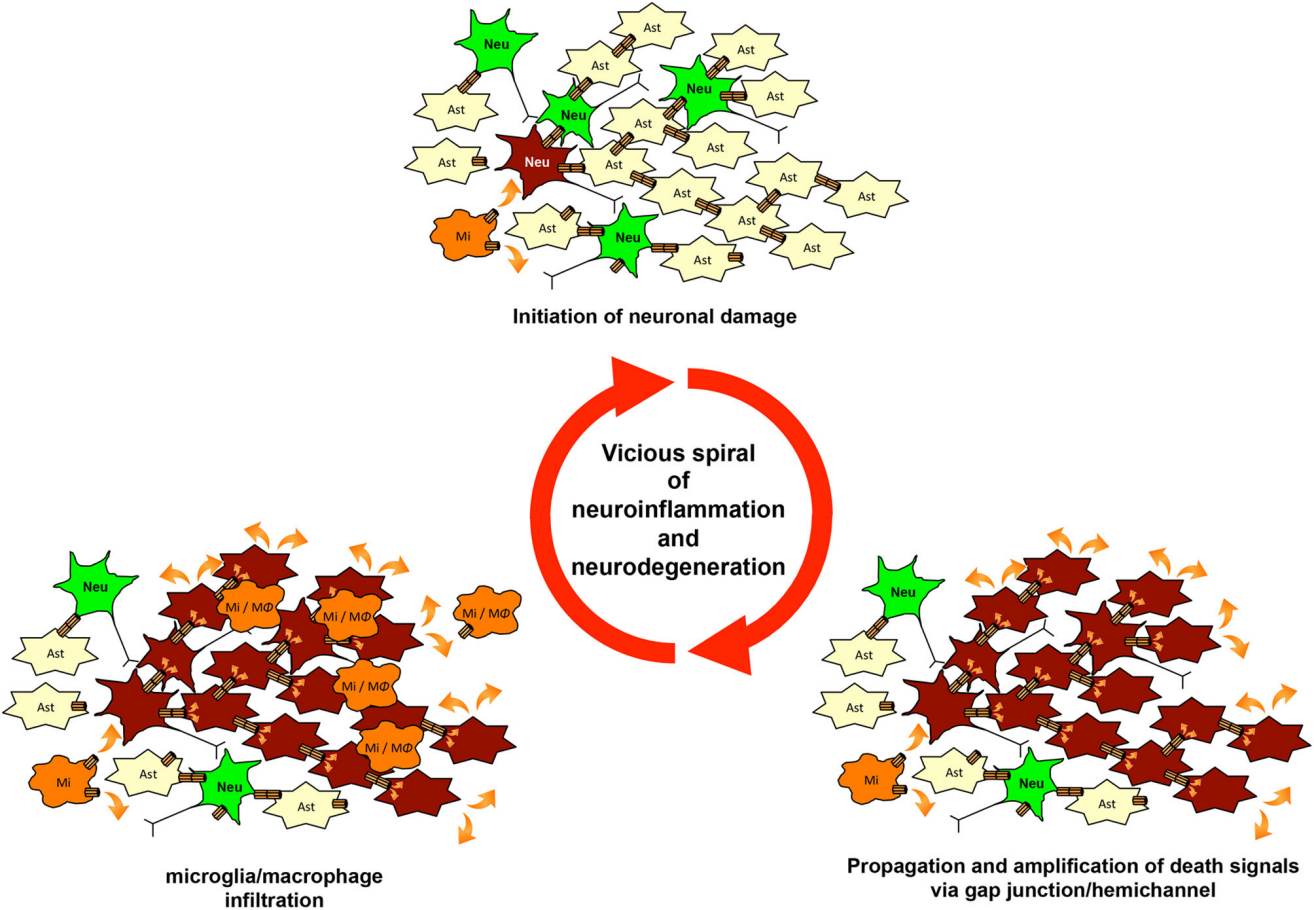
**The vicious spiral of neuroinflammation and neurodegeneration mediated by gap junctions and hemichannels**. Microglial glutamate release via hemichannels initiates neuronal damage. Then, waves of death signals are propagated and amplified via glial and neuronal gap-junction communication. Chemoattractants from damaged cells induce infiltration by microglia and macrophages. These infiltrating cells initiate further neuronal damage and enlarge the lesion of neuroinflammation and neurodegeneration. This vicious spiral of neuroinflammation and neurodegeneration may be involved in the progression of various neurologic diseases.

As mentioned above, glial gap junctions play an important role in maintenance of homeostasis in the CNS under the physiological conditions. These structures, however, also contribute to the initiation and propagation of pathologic conditions (Orellana et al., 2009). Stroke and trauma provide examples that illustrate this mechanism. Ischemia or contusion leads to a rapid decrease in intracellular oxygen levels and subsequent reduction in ATP synthesis, resulting in eventual cell death (Kalogeris et al., 2012). Injured cells contain toxic ions and molecules at high concentrations (e.g., Ca^2+^, K^+^, ROS, and NO). These toxic molecules are propagated from injured cells to healthier cells through gap junctions. Ischemic conditions also induce uncoupled hemichannels to open, leading to paracrine transfer of toxic molecules (Thompson et al., 2006; De Vuyst et al., 2007). These waves of death signals activate astrocytes and microglia, inducing the release of toxic molecules including glutamate, ROS, NO, and pro-inflammatory cytokines and chemokines. This vicious amplification spiral of signaling could worsen neuroinflammation by recruiting leukocytes and increasing the lesion area (Orellana et al., 2009) (Figure 3). Moreover, gap junction and hemichannel blockers have exerted therapeutic effects in experimental models of stroke and spinal cord injury (Rawanduzy et al., 1997; Frantseva et al., 2002; De Pina-Benabou et al., 2005; Takeuchi et al., 2008a; Tamura et al., 2011; Huang et al., 2012; Umebayashi et al., 2014).

Abnormal expression of glial connexins has been observed in the inflamed lesions in multiple sclerosis (MS) and an animal model of this disease, experimental autoimmune encephalomyelitis (EAE). In particular, downregulation of oligodendrocytic Cx32 and Cx47 and astrocytic Cx43 have been observed in the active lesions of MS patients and EAE mice (Brand-Schieber et al., 2005; Eugenin et al., 2012; Markoullis et al., 2012). Expression levels of Cx47 and Cx32 were upregulated during remyelination, but downregulated in the relapsing phase, and Cx32 deletion resulted in exacerbates symptoms in EAE, specifically increased demyelination and axonal loss (Markoullis et al., 2012). Whereas mice lacking astrocytic expression of Cx43/Cx30 exhibited white-matter vacuolation and hypomyelination, the severity of EAE in these animals was similar to that in wild-type mice (Lutz et al., 2012). Therefore, oligodendrocytic expression levels of Cx32 and Cx47 appear to be associated with the degree of damage and remyelination, whereas astrocytic expression levels of Cx43 do not. However, recent studies showed that a loss of Cx43 in astrocytes precedes demyelination in the MS-related disorders neuromyelitis optica and Balo's disease (Matsushita et al., 2011; Masaki et al., 2012), suggesting that the temporal expressional pattern of astrocytic Cx43 plays a significant role in the disease process.

Accumulating evidence has also implicated neuroinflammation, including gliosis by activated astrocytes and microglia, in the pathogenesis of such neurodegenerative diseases as HIV encephalopathy, AD, PD, and ALS (Glass et al., 2010; Valcour et al., 2011). Microglial activation followed by astrocytic activation is the earliest pathologic feature in the pre-symptomatic phases of these diseases. Our recent studies have shown that activated microglia release excess glutamate through Cx32 hemichannels, resulting in excitotoxic neuronal death (Takeuchi et al., 2006, 2008a; Yawata et al., 2008). Furthermore, microglia-derived glutamate and pro-inflammatory cytokines induce dysfunction of gap junctions and hemichannels in astrocytes (Kielian, 2008), thereby potentially disrupting CNS homeostasis. On the other hand, reactive astrocytes neighboring amyloid β (Aβ) plaques in the brains of AD patients expressed elevated levels of Cx43 and Cx30 (Koulakoff et al., 2012). Aβ peptide induces the release of glutamate and ATP via uncoupled hemichannels in microglia and astrocytes, leading to neuronal death (Orellana et al., 2011). Corroborating this observation, blockade of gap junctions/hemichannels improved memory impairment in a mouse model of AD (Takeuchi et al., 2011). Recent studies also revealed that astrocytic gap junctions/hemichannels are involved in the disease progression of HIV encephalopathy (Eugenin and Berman, 2013; Orellana et al., 2014). PD animal models (MTPT-treated mice and rotenone-treated rats) exhibited upregulation of astrocytic Cx43 expression in affected areas (Rufer et al., 1996; Kawasaki et al., 2009), and a gap junction/hemichannel blocker ameliorated the disease symptoms of a PD mouse model (Suzuki et al., 2014). A recent report revealed that α-synuclein directly binds Cx32, and that overexpression of α-synuclein suppresses the activity of Cx32 in the SH-SY5Y dopaminergic neuroblastoma cell line (Sung et al., 2007). Other studies have shown that microglia and astrocytes are determinants of disease progression in ALS (the non-autonomous neuronal death hypothesis) (Boillee et al., 2006; Yamanaka et al., 2008). Activation of microglia and astrocytes is associated with elevated expression of gap junctions and hemichannels (Cui et al., 2014). In fact, treatment with a gap junction/hemichannel blocker ameliorated disease progression in a mouse model of ALS (Takeuchi et al., 2011). Juvenile neuronal ceroid lipofuscinosis (JNCL) also shows the activation of microglia and astrocytes preceding neuronal loss (Pontikis et al., 2005; Xiong and Kielian, 2013), and treatment with a gap junction/hemichannel blocker attenuated the disease symptoms of a JNCL mouse model (Burkovetskaya et al., 2014). Few reports, however, have focused on the expression profiles and functions of connexins in these diseases. Further studies are needed to elucidate the precise role of glial connexins in the pathogenesis of these diseases.

## Conclusions

A growing body of evidence has demonstrated the pathologic roles of gap junctions and hemichannels in various neurologic diseases. For example, dysfunction and dysregulation of gap junctions and hemichannels in glial cells contribute to neuroinflammation in the CNS, which results in neuronal damage (a situation in which glial cells are “bad neighbors” of neurons) (Block et al., 2007). Despite recent progress in elucidating the pathological roles of gap junctions and hemichannels, many challenges remain, due in part to technical limitations. For instance, few high-quality antibodies against each connexin are available for immunostaining and immunoblotting. Moreover, reagents that are commonly used to block connexin channels are not specific for those channels. In fact, connexin channel blockers such as glycyrrhetinic acid, its derivative carbenoxolone, niflumic acid, and octanol also block pannexin channels. Although the most specific gap junction and hemichannel blockers currently available are mimetic peptides with sequences very similar to that of the extracellular loop of connexins, recent studies showed that mimetic peptides specific for Cx32 (^32^gap 24 and ^32^gap 27), Cx43 (^43^gap 27), or Panx1 (^10^panx1) non-specifically block both connexins and pannexins (Wang et al., 2007). Although aptamers and siRNA may be used as blockers for specific connexins (Knieps et al., 2007; Xu et al., 2014), they still have a problem of the blood–brain barrier penetration. The heterogeneity of gap-junction and hemichannel configurations (Figure 2) and the ability of various connexins to compensate for the loss of other isoforms (e.g., in connexin-knockout studies) also complicate analysis of this system. Although EGFP-tagged connexins have facilitated live-cell imaging, tagging and/or overexpression of connexins in cultured cells often produce abnormally large gap-junction plaques (Lopez et al., 2001; Gaietta et al., 2002; Hunter et al., 2003). Moreover, tagging the amino-termini of connexins results in non-functional channels, whereas tagging the carboxyl-termini alters the properties of the channels (Bukauskas et al., 2000; Contreras et al., 2003). Therefore, future investigations should attempt to elucidate the spatiotemporal expression profiles of connexin isoforms under pathological conditions in the CNS; this work will require development of specific blockers and tracers for each connexin isoform, hemichannel, and gap junction. Understanding the precise pathologic roles of gap junctions and hemichannels may lead to new therapeutic strategies against multiple chronic neurodegenerative diseases.

### Conflict of interest statement

The authors declare that the research was conducted in the absence of any commercial or financial relationships that could be construed as a potential conflict of interest.
